# Mitochondrial Redox Hubs as Promising Targets for Anticancer Therapy

**DOI:** 10.3389/fonc.2020.00256

**Published:** 2020-02-28

**Authors:** Luigi Ippolito, Elisa Giannoni, Paola Chiarugi, Matteo Parri

**Affiliations:** Department of Experimental and Clinical Biomedical Sciences, University of Florence, Florence, Italy

**Keywords:** mitochondria, ROS—reactive oxygen species, tumor microenviroment, anti oxidant, anticancer activity, redox targeting

## Abstract

Mitochondria play multifaceted roles in malignant tumor progression. Beyond their bioenergetic role, mitochondria are essential for providing malignant cells a higher plasticity to face the harsh environmental conditions. Cell-autonomous metabolic deregulation of cancer cells, or metabolic adaptation to microenvironmental cues (lack of nutrients, stromal supply, hypoxia, etc.), represent the triggering event of mitochondria overexploitation to orchestrate nutrient sensing and upload, signaling, and redox circuits. As readout of their higher function, mitochondria produce high amounts of reactive oxygen species (ROS) that are functional for multiple signaling networks underlying tumor proliferation, survival, and metastatic process. To compensate for the higher rate of mitochondrial ROS production, cancer cells have evolved adaptive mechanisms to increase their antioxidant systems and to address ROS activating pathways useful for the tumor cell adaptation to environmental changes. As these properties are critical for cancer progression, mitochondrial ROS have recently become an attractive target for anti-cancer therapies. We discuss how understanding of mitochondrial function in the tumor-specific generation of ROS will impact on the development of novel redox-based targeted therapeutic strategies.

## Introduction

### Mitochondrial ROS: Source and Regulation in Cancer

Tumor cells exhibit metabolic plasticity that provides them with a selective advantage to face harsh microenvironmental conditions (e.g., hypoxia, acidosis, low nutrients availability). Beyond the Warburg effect (i.e., high upload of glucose and its conversion into lactate nevertheless of oxygen availability), tumor cells can use several nutrients to fuel mitochondrial metabolism and support growth. Indeed, within the tumor cell, mitochondria represent dynamic organelles that orchestrate a variety of signals, able to meet and finely adjust the fluctuating metabolic needs of the cell. In this scenario, mitochondria promote the anabolic and catabolic machinery of a tumor cell by driving fatty acid oxidation (FAO), TCA cycling, the electron transport chain (ETC) and to provide intermediates to synthesize macromolecular building blocks such as amino acids, lipids, nucleotides, and iron sulfur clusters, as well as reducing agent (NADPH) for their antioxidant systems, respectively.

Reactive oxygen species (ROS) represent highly reactive molecules resulting from oxygen (O_2_) including radicals and non-radicals, produced inside the cells through a high metabolic activity consuming oxygen. Therefore, at higher levels, ROS are responsible for the damage of most of cellular macromolecules, whereas they exhibit signaling role at moderate levels. As by-product of intense oxidative phosphorylation (OXPHOS), mitochondria specifically generate mitochondrial ROS (mROS). ETC complexes I and III by leaking free electrons lead to a mono-electronic O_2_ reduction to superoxide (O_2_∙-), which is readily reduced by superoxide dismutases (SODs) to hydrogen peroxide (H_2_O_2_). The current idea is that cancer cells manifest a peculiar pattern of high ROS levels than non-transformed cells following the action of oncogenes, the loss-of-function of most tumor suppressor genes, the deregulation of metabolism, mitochondrial dysfunction, inflammatory burst, or genotoxic stress (1) and this is counteracted by a strong and highly regulated antioxidant machinery. mROS are counteracted by a compartmentalized antioxidant apparatus not linked to the cytosolic one (2), with mitochondria using their own antioxidant enzymes, such as glutathione reductases, catalases, peroxidases, and other NADPH-generating sources.

The main transcriptional response underlying the antioxidant action in tumor cells is due to the activation of nuclear factor (erythroid-derived 2)-like 2 (NRF2) (3). NRF2 is tightly and positively regulated upon the inhibition of Kelch-like ECH-associated protein 1 (KEAP1) and thus NRF2 stabilization promotes the transcription of genes coding for antioxidants enzymes. NRF2-KEAP1 canonical pathway is regulated by the high ROS levels increased within the cell. In this setting, oxidants induce the modification of specific cysteines on Keap1 structure leading to the loss of basal cytosolic sequestration and the ubiquitination of Nrf2. This results in Nrf2 translocation into the nucleus to induce the transcription of antioxidant genes (3). Also, a ROS-independent Nrf2 activation has been found upon the activity of p62, a marker of cellular stress. p62 binds to Keap1, thereby preventing Keap1-mediated trapping of Nrf2, thereby inducing Nrf2 stabilization and activation (4). Balancing ROS initiation and ROS scavenging allows cancer cells to achieve and manipulate ROS within a certain sub-toxic tumorigenic range. Enhancement or disruption of antioxidant capacities can be beneficial or detrimental for tumor cells, respectively, as it can prevent or not ROS from reaching cytotoxic levels. Consequently, the finely-tuned ROS generation and scavenging are two aspects fundamental to cancer cells since ROS-mediated cell signaling can significantly impact on a wide range of cellular pathways, including tumor initiation and proliferation, survival, de-differentiation, and metastatization.

## Effects of Cell-Autonomous Mitochondrial ROS on Tumor Progression

Inactivation of tumor suppressor genes, as well as activity of oncogenes and mitochondrial DNA (mtDNA) mutations, are cell-autonomous mechanisms, somehow regulated by and regulating tumor ROS. However, these mechanisms underlying activation of mitochondrial ROS generation can be associated to a rewiring of the mitochondrial metabolism of tumor cells [e.g., MYC/KRAS or MYC/ERBB2 ablation in breast and pancreas cancer cells (5–7)]. Such metabolic remodeling reflects the genetic and environmental landscape of a cancer cell in given circumstances.

### Effect of mROS on Tumor Initiation and Progression

Mitochondria may contribute to malignant phenotype as mROS can lead to DNA aberrations and activation of oncogenic pathways. Mutations in mtDNA-encoding ETC have been investigated in several tumor models as prone to confer a selective advantage in tumor initiation. Indeed, mROS derived from mutated complex I is crucial for the tumor formation *in vivo* (8). Conversely, loss-of-function mutations of mitochondrial transcription factor A (TFAM), which participates to the replication of mitochondrial genome, are incompatible with tumor formation *in vivo* (9), while heterozygosity for TFAM is crucial for a ROS-dependent intestinal tumorigenesis (10). Interestingly, the seminal study by Ishikawa et al. explored the pro-metastatic role of exogenous mtDNA acquired by recipient cancer cells with low propensity to metastatize. The acquisition of mtDNA confers high metastatic potential *via* the overproduction of mROS derived by mtDNA-transmitted complex I mutation and the up-regulation of nuclear genes such as HIF-1α, VEGF, and MCL-1 (myeloid leukemia cell protein-1) involved in metastasis (11). However, ROS-mediated DNA damage promotes genomic instability in gliomas models, particularly by mediating the deletion or mutation of tumor suppressor genes such as TP53, a driver for maintaining functional antioxidant defenses (12). Finally, subsets of breast cancer cells derived from primary tumors have been shown to display differential mROS content. High mROS-loading cells activate mitochondrial unfolded protein response (UPR^mt^) and its activation regulates cytoprotective mechanisms in a SIRT3-dependent manner, resulting in mitochondrial rewiring as well as in resistance to subsequent oxidative stress (*mitohormesis*, i.e., the activation of cytoprotective mechanisms such as the unfolded protein response, important for improving the cellular resistance to mitochondrial stresses). As phenotypic readout, mitohormesis primes this subpopulation to be highly metastatic compared to low mROS-producing cells, as metastasis, angiogenesis, and cell migration gene sets are positively enriched in UPR^mt−HIGH^ patients (13).

### Effect of mROS on Tumor Energy Metabolism

Furthermore, mutations of specific TCA cycle enzymes such as succinate dehydrogenase (SDH), fumarate hydratase (FH), and isocitrate dehydrogenase (IDH) may dramatically alter cell bioenergetics and consequently the ROS production. SDH complexes—(e.g., Sdhb, SdhC, and SdhD), when inactivated, have been addressed as factors leading tumorigenesis via mROS. Particularly, the pharmacological or genetic impairment of specific subunits of SdhB, increases superoxide production, increases HIF-α stabilization in an ROS-dependent manner, and this has a positive impact on the growth rates *in vitro* and *in vivo* (14). FH alterations observed in models of hereditary leiomyomatosis and renal cell cancer promotes the accumulation of fumarate able to load a succination reaction on the glutathione to produce the metabolite succinated glutathione (GSF) and Keap1 (15). Thus, GSF acts as a NADPH-consuming metabolite used by glutathione reductase, thus reducing antioxidant capacity and resulting in increased mROS that, maintained in homeostatic levels by the simultaneous activation of Nrf2, promote tumorigenic signaling. Also, alterations in IDH1/2 may cause not only the complete loss of wild-type enzymatic functions, but also an increase in ROS levels due to the impaired action of NADPH and GSH (16). Overall, choked TCA cycle and/or OXPHOS are functional for ROS generation. Mechanistically the alterations in these key TCA cycle enzymes provoke metabolic perturbations (e.g., succinate and/or fumarate accumulation) leading to signaling cascades such HIF1 activation (17, 18). Besides these mechanisms involving TCA intermediates-dependent inhibition of HIF-degradation-mediating enzymes such as prolyl hydroxylases, also ROS derived by the overexploitation of mitochondria can trigger HIF1 stabilization. So far, while mROS generation from complex I is predominantly located into mitochondrial matrix, complex III is capable of producing ROS able to act as signaling molecules upon diffusion into cytosol. These mROS can oxidize some cysteines in prolyl hydrozylase 2 (PHD2), affecting its enzymatic activity and thus allowing for the stabilization and the subsequent activation of HIF1α and HIF-induced genes, respectively (19).

### Effect of mROS on Tumor Stemness

As tumor expansion is a feature strictly related to the ability of a malignant cell to display tumor-initiating and de-differentiating potential (stemness), regulation of ROS levels is useful for cancer stem cells (CSCs) to elicit their hallmark features. However, CSCs are highly heterogeneous in their metabolic and redox profiles. Different mitochondrial exploitation and consequently mROS generation has been addressed in different models. Accordingly, in liver CSCs the stemness marker NANOG, upon activation of Toll-like receptor 4 (TLR4)-E2F1 axis, negatively impacts on mitochondrial respiration and ROS generation (20). Similarly, in acute myeloid leukemia, mROS^low^ CSCs are paradoxically OXPHOS-dependent and overexpress Bcl-2. Interestingly, Bcl-2 inhibition eradicates the quiescent stem cells by increasing mROS. Conversely, ovarian CSCs privilege OXPHOS metabolism and mROS production sustains this phenotype (21). Additionally, in other models of CSCs, mROS due to high lipid catabolism trigger the activation of MAPK as well as of epithelial-mesenchymal transition (EMT), consequently potentiating cancer invasion and metastasis (22). Notably, in breast cancer, a high plasticity in determining cellular stem-like states results in two distinct metabolic profiles of stem cell sub-populations. While mesenchymal-like breast CSCs undergo a typical Warburg metabolism but repress ROS to sustain their stemness status, the epithelial-like ones are highly respiratory and show high OXPHOS-dependent ROS. Also, pancreatic cancer stem cells surviving to KRAS ablation rely on mitochondrial metabolism and show increased mROS levels (5). Furthermore, CSCs relying on mitochondrial metabolic rewiring frequently are responsible of tumor relapse and acquire resistance to chemotherapies and other treatments (23). Accordingly, the levels of mitochondria-derived ROS are presumably functional for the maintenance of resistant phenotype.

### Effect of mROS on Tumor Chemosensitivity

The modulation of mitochondrial redox state exerts different roles in the chemoresistance scenario, thereby reflecting the high heterogeneity and plasticity of cancer cells. Indeed, negative regulation of mitochondrial gene transcription by mitomiRNA-2392 is coupled with increased mROS levels as well as chemosensitivity profile of resistant squamous cell carcinoma cells (24). Also, persistent oxidative stress leads to the accumulation of promyelocytic leukemia protein (PML)-nuclear bodies in ovarian carcinomas, resulting in activation of the transcriptional co-activator PPARγ coactivator-1α (PGC-1α). Active PGC-1α-mitochondrial respiration axis results in high mROS content in these cells as well as alterations in iron homeostasis. In keeping, high ROS levels represent a key element in the modulation of the chemosensitivity of high-OXPHOS cancer cells. Indeed, high-OXPHOS cells exhibit increased response to conventional chemotherapies, and PML silencing is able to reduce the sensitivity of high-OXPHOS cells to a ferroptosis drug such as ironomycin, thereby showing that this sensitivity is linked to PML and OXPHOS status (25). Also, MYC activation cooperatively with myeloid cell leukemia-1 protein has been reported to sustain the mitochondrial upgrading in terms of biogenesis and respiration of chemotherapy-resistant CSCs in triple negative breast cancer. The increase of mitochondrial respiration and mROS production lead to HIF1-dependent expansion of resistant CSCs (26). Similarly, resistant clones of pancreatic CSCs—showing OXPHOS dependency and high antioxidant properties—emerged during OXPHOS inhibition treatment rely on MYC activation as a metabolic switcher, by regulating the transition from a PGC-1α-dependent strong mitochondrial activity toward an intermediate glycolytic/respiratory phenotype culminating in high mROS content (27).

## Effects of Cell Non-Autonomous Mitochondrial ROS on Tumor Progression

It has been largely recognized that mitochondria exhibit a multifaceted role in triggering and orchestrating cellular responses to environmental stimuli and signals derived by hypoxic conditions and/or stromal cells, thereby regulating, among others, cell non-autonomous mROS production. The microenvironment in the primary tumor primes the cancer cells to remodel their metabolism to permit the escape from it and the metastatization in distant organs.

### Effect of mROS on Tumor Metastatization

Metastatization depends on the ability of tumor cell to survive detachment from the extracellular matrix and subsequently undergo intravasation. Loss of attachment provokes mitochondrial alterations in cancer cells. Indeed, cells detached from the matrix undergo metabolic reprogramming by upregulating pyruvate dehydrogenase (PDH) kinase, thereby attenuating the flux of glycolytic carbon (i.e., pyruvate) into mitochondrial oxidation, ultimately affecting the mitochondria functionality and ATP levels (28, 29). Recently, anchorage-deprived tumor cells have been shown to rely on a metabolic phenotype based on reductive carboxylation of glutamine, which supports the production of mitochondrial NADPH useful to combat mROS (30). However, the induction of a hypoxic-dependent cell clustering induction has been shown to mediate mitophagy able to clear damaged mitochondria and limit mROS burst, thereby supporting tumor cell survival during extracellular matrix detachment and metastatic spread (31). The management of mitochondrial ROS levels is a key feature of acute myeloid leukemia, where the protein kinase-ε activation controls the content of mROS by impacting on some ROS-buffering enzymes, such as thioredoxin and glutathione synthetase, thereby promoting the tumor progression *in vivo* (32). Importantly, modulation of antioxidant defense against mROS has been reported to be crucial for metastatic cancer cells. Indeed, it has been reported that one-carbon metabolism maintains the mitochondrial redox homeostasis during hypoxic conditions. Indeed, mitochondrial serine hydroxymethyltransferase (SHMT2) is induced in hypoxic conditions and its activity results in a high NAPDH-dependent mROS balance, as the methyl donor methylene-tetrahydrofolate can be oxidized through the folate metabolic enzymes to generate NADPH, which is important for maintaining cellular redox balance (33). Also, circulating and secondary sites-engrafted cancer cells displayed higher levels mROS than those isolated from the primary melanomas. However, upon metastatic dissemination, melanoma cells rely on folate pathway for the capacity to exploit folate-derived NADPH to neutralize oxidative stress (34). In apparent contrast, Porporato et al. have shown that metastatic colonization is sustained by superoxide derived by dysfunctional mitochondria. In particular, mROS are able to target and activate Src-Pyk molecular axis underlying the migration machinery of cancer cells (35) and pharmacological scavenging of mitochondrial superoxide prevent metastatic dissemination. In this scenario, the mitochondrial reprogramming is often associated with the activation of PGC-1α, a transcriptional regulator mitochondrial biogenesis and oxidative metabolism. However, it is reported that PGC-1α increases mitochondrial respiration in melanoma cells and, at the same time, helps them to manage the oxidative stress by orchestrating the activation of ROS-detoxification systems such as glutathione (36) and thus sustaining tumor growth. Conversely, PGC-1α overexpression impairs prostate and renal cancer progression (37, 38). Thus, the role of PGC-1α in the management of both the mitochondrial dynamics/functionality and redox biology (i.e., upregulation of SOD2, Nrf2, and GSH)—beneficial or detrimental for the tumor progression—seems to be paradoxical; however, it reflects the tumor metabolic heterogeneity and new insights on the impact of the spatio-temporal tuning of fluctuations of mROS levels on cancer cell state need further investigations.

Metastatic dissemination and tumor formation and growth are well-known hallmarks enabled and sustained through contributions from multiple repertoires of stromal cell types. Particularly, rewiring of mitochondrial metabolism has been extensively affected by tumor stroma (39). By expanding Ishikawa's findings, it has been shown that *in vivo* horizontal transfer of intact mitochondria from stromal compartment in the host animal to the mitochondria-depleted melanoma tumor results in a renewed OXPHOS and tumorigenic capacity in the mitochondria-deficient recipient cells (40). The incorporation of host mitochondria can reasonably have an impact on the levels of mROS, newly exploitable as tumorigenic signals. In addition, a metabolic cross-talk driven by lactate derived by cancer-associated fibroblasts promotes a mitochondrial exploitation in prostate carcinoma cells. Consequently, lactate-driven mROS burst is essential for EMT engagement and invasiveness of these stromal-reprogrammed cancer cells. Also, mROS are able to oxidize Src and pyruvate kinase-M2, key molecules in the establishment and maintenance of the metabolic loop (41, 42). At the same time, mitochondrial redox dynamics underlies the shaping of the tumor microenvironment promoted by stromal cells. Mitochondria dysfunction in fibroblasts induced by long-term radiation induces mROS increased levels. These activate TGFβ signaling which in turn mediate the expression of α-SMA, a key marker of the fibroblast reactivity indispensable for the tumor growth (43).

### Effect of mROS on Tumor Immune Environment

The control of mitochondria-derived ROS has been increasingly explored as a driver of the recruitment and the activation of immune cells in tumor environment. It has been observed that cancer patients show inactive subsets of immune cells and their neutrophils show higher OXPHOS metabolism (44). Indeed, neutrophils maintain NADPH-oxidase-dependent ROS production, under glucose starvation, through balancing with NADPH levels supplied by fatty acids oxidation. This strategy is important for neutrophils to overcome the nutritional limitations occurred in the glucose-deprived tumor microenvironment, where mitochondria is exploited and functional for promoting neutrophil activation and the inhibition of the adaptive anti-tumor immune responses (44). Also, regulatory T cells (Tregs) are committed to survive and proliferate in such a hostile milieu like tumor microenvironment because they display and exploit both glycolysis and fatty acid synthesis/oxidation, allowing them to predominate over CD4/CD8^+^ T cells mainly relying on the glycolysis (45). In keeping, the suppression of OXPHOS through complex I inhibitor such as metformin is detrimental for tumor-infiltrating Treg cells differentiation from T naïve cells, probably acting on mROS content (46). To note, Treg cells require a functional mitochondrial complex III to maintain their suppressive function, suggesting a fascinating role of mROS in the metabolic and epigenetic landscape of Treg cell compartment (47). Interestingly, ovarian-cancer-infiltrating Treg cells showing high OXPHOS dependency produce higher amounts of mitochondrial ROS and undergo apoptosis, thereby mediating the local production of immunosuppressive molecules able to act on other immune cell types and, consequently, a higher immune escape of tumors and failure of the clinical approach involving Treg depletion (48). To note, high exploitation of glucose by cancer cells results in a glucose-deprived microenvironment, leading to the establishment of a metabolic competition between tumor and immune cells (49), resulting in a T cell mitochondrial dysfunction and repression (50). In such metabolic scenario, CD8+ tumor-infiltrating cells have been characterized to be unable to efficiently uptake glucose and to have hyperpolarized, fragmented mitochondria generating large amounts of mROS, otherwise required for CD4+ T cell activation (51). These defects in glucose uptake and glycolysis as well as in mitochondrial dynamics and function contribute to the failure of CD8+ T cell activation and exhaustion state in the microenvironment, where the renal carcinoma cells are highly glucose- consuming and depriving (52). Accordingly, in glucose-deprived conditions, tumor cells utilize mitochondria as glucose sensor by increasing mROS-dependent oxidative stress and post-translationally regulating the expression of monocarboxylate transporter 1, whose functional activity of lactate transport is dependent on pH gradient. This response as result of mitochondrial dysfunction drives the migration of cancer cells conferring them the ability to evade a glucose-depleted environment (53). mROS as well as other mitochondrial-associated metabolites critically impact on the effector pro- and anti-inflammatory functions of M1 and M2 macrophages, respectively (17). Indeed, M1 and M2 macrophages differ significantly in the major pathways of energy utilization and provision. However, mROS function is controversial in these cells. It has been shown that complex I-derived ROS are bactericidal and activate the inflammosome in M1 macrophages (54). On the other hand, mROS activate the NF-κB-dependent recruitment and polarization of M2 macrophages (55), although in non-tumoral circumstances. It is likely that, in the tumor microenvironment, macrophages undergo mitochondrial adaptations according to the nutrient availability (and mROS levels change, accordingly). For example, lactate, highly enriched in the tumor environment, shows a polarizing effect on M1 macrophages toward the M2 pro-tumor phenotype (56) and the HIF1-associated signaling cascade is presumably modulated also upon a renewed lactate-dependent mitochondrial redox state. In keeping, high levels of mROS promote pro-invasive signature of melanoma-associated M2 macrophages by regulating their tumor necrosis factor α secretion (57). Finally, in the complex immune environment, the dendritic cells (DCs) are responsible for the antigen presentation, which is crucial for the activation and the recruitment of CD8^+^ anti-tumor T-cell population. Recently, the reduced mROS levels in DCs impair their ability in cross-presentation and, consequently, in the culminating anti-tumor effect mediated by CD8^+^ T-cell response *in vivo* (58), suggesting a potential role of mROS in modulating protective immunity against tumor.

**Figure 1:**
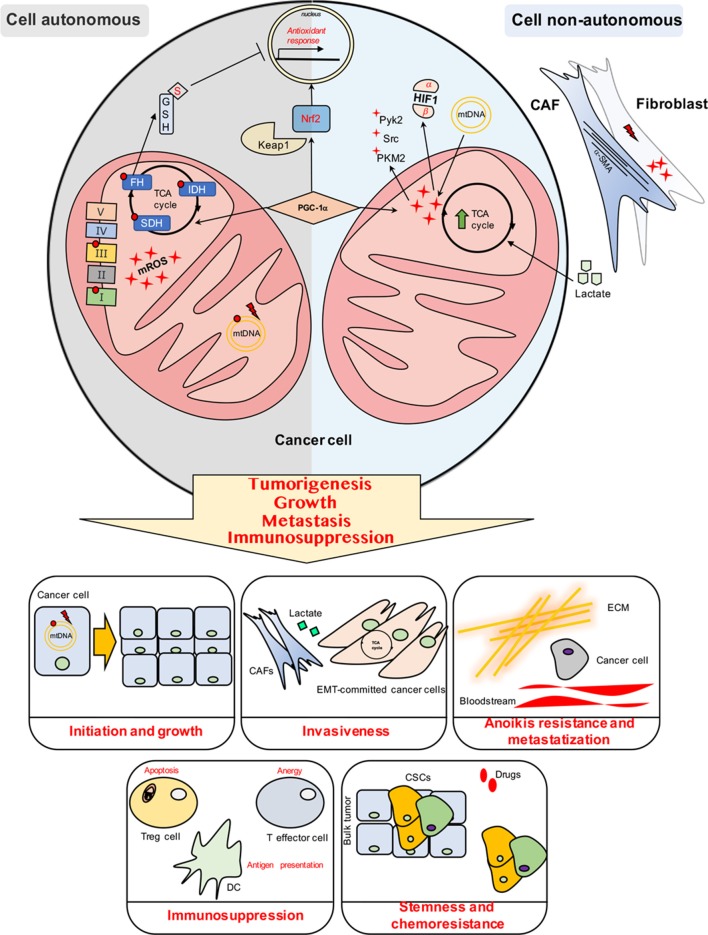
Cell autonomous and cell non-autonomous mechanisms impact on the mitochondrial ROS (mROS) production and management in cancer cells.

Mitochondrial redox homeostasis crucially impacts on tumor cells behavior and regulates several hallmarks of cancer. Altered mitochondrial metabolism due to genetic dysfunction and/or microenvironmental signals provides a scenario where cancer and cancer-accessory cells need to exploit and maintain a specific threshold for compartmentalized mROS levels to orchestrate mROS-dependent signaling machinery during the tumor progression (Figure 1). However, the compartmentalization of the ROS generated and localized in the mitochondria in tumor and/or stromal cells provides a challenging issue to target and reduce mROS by an anti-tumoral perspective.

## Targeting Mitochondrial Redox Capacity

The several crucial functions of mitochondrial redox activity in tumor progression and in the advanced stages of cancer, making them a suitable choice for anti-tumor treatment. Methods that address mitochondrial metabolism has been demonstrated to be active in various clinical cancer researches and extensively studied in Weinberg and Chandel (59). Despite the fact that some tumor cells increase their production of ROS, other tumor cell lines involve antioxidant systems to ensure that ROS levels do not attain a dangerous point. It is not unexpected that the cure of cancer with antioxidants has been favorable in a few studies, while unsuccessful in others. Vitamin C increases the power of the anti-proliferative role of doxorubicin in breast tumor (60), whereas high vitamin D levels are pertinent with a higher survival rate in patients with colorectal cancer (61). Therapy with carotenoids could increase fatality in breast cancer patients (62) whereas treating the same patients with vitamin C and E has been associated with a good overall survival (62). It may be deduced that the dietary antioxidants fail because they do not attain the restricted ROS produced by the mitochondria (59). The dual behavior of mROS shows that they can both assist or limit tumor initiation and growth. Therefore, mitochondrial antioxidant and pro-oxidant strategies have been considered for anti-cancer treatments (Table 1).

**Table 1:** Drugs targeting mitochondrial redox capacity.

**Drug**	**Mechanism of action**	**References**
MitoQ	Antioxidant that target mitochondria. The antioxidant properties of quinone are combined with the lipophilic cation TPP.	(63, 64)
MitoTEMPO	Antioxidant that target mitochondria. The antioxidant properties of piperidine nitroxide (Tempo) are combined with the lipophilic cation TPP.	(63–65)
SkQ1	Antioxidant that target mitochondria. The antioxidant properties of plastoquinone are combined with the lipophilic cation TPP.	(63, 66)
Phenformin	Mitochondrial respiration inhibitor that block the mitochondrial complex I.	(67)
Mito-metformin	Mitochondrial targeted metformin. Mitochondrial respiration inhibitor that block the mitochondrial complex I.	(67, 68)
MitoVitE	This compound is made by α-tocopherol attached to TPP. Inhibitor of complex I-mediated mitochondrial respiration.	(63–65)
MitoChM	Mitochondria-targeted vitamin E analog. Mitochondria-targeted antioxidant.	(69)
MitoChM-Ac	MitoCh analog. Mitochondria-targeted antioxidant.	(69)
Mito-CP	Inhibits complex I-mediated mitochondrial respiration.	(70)
Mito-CP-Ac	Mito-CP analog. Inhibits complex I-mediated mitochondrial respiration.	(70)
Menadione-Ascorbate	Mitochondria-targeted pro-oxidant.	(71)
B-phenylethylisothiocyanate (PEITC)	Mitochondria-targeted pro-oxidant.	(71)
ME-344	Strong mitochondrial OXPHOS complex I inhibitor.	(67)
MitoVES	α-tocopheryl succinate with improved efficacy. Block the mitochondrial complex II, leading to generation of ROS.	(72)
Dichloroacetate (DCA)	Antioxidant that target mitochondria.	(73)

### Mitochondrial Antioxidant Strategies

Targeting specific mROS with the improvement of the production of antioxidants targeted to specific subcellular compartments (63) represent potential advances for antioxidant cancer therapies. Many approaches of mitochondria-directed pharmaceutical administration have newly been developed. One of these methods utilize de-localized lipophilic cations (DLCs), which are specific for mitochondrial negative charge matrix and pass the membranes of mitochondria (64, 65). Specific antioxidants that are targets of mitochondria (MTAs), including MitoQ, MitoTEMPO, and SkFQs, are noted to pass all biological membranes and collect inside mitochondria easier than their non-targeted parent antioxidants [due to its link to triphenylphosphonium (TPP)], rendering them effective in guarding against mitochondrial oxidative damage (64).

#### MitoQ

One of the most studied antioxidant molecules that target mitochondria is MitoQ (mitoquinone). It consists of TPP covalently liked to the ubiquinone moiety of the endogenous antioxidant coenzyme Q10 (CoQ10) by a ten-carbon aliphatic chain. MitoQ distinctly reduces proliferation of diverse melanoma xenograft models of carcinogenesis (35). MitoQ, impedes the advancement of spontaneous metastasis of tumorigenic MDA-MB-231 human breast cancer cells embedded orthotopically, in the mouse mammary fat pad (69).

#### MitoTEMPO

Another TPP derivative is MitoTEMPO, however it is associated with the stable piperidine nitroxide radical TEMPO, which obtains an electron from hydroxylamine a strong radical scavenger. MitoTEMPO can also function as a cytosolic superoxide dismutase (SOD) mimetic, which transforms superoxide into water and enables ferrous iron to be oxidized into ferric iron. MitoTEMPO inhibits melanoma cell growth, reduces melanoma cell viability, and induces apoptosis, but does not affect non-malignant skin fibroblasts (35, 69). Diminished mROS production in mitoTEMPO treated-melanoma cells modifies cell signaling mediated by mROS-sensitive Akt, ERK1/2, and HIF1-α and decreases xenograft growth in mice (74).

#### SkFQs

A different group of mitochondria targeted antioxidants named “SkFQs” was created by Skulachev et al. utilizing plastoquinone to change the ubiquinone antioxidant moiety of MitoQ (66). One of the greatest investigated “Sk” molecules is the compound named SkQ1, which is a TPP derivative associated with plastoquinone itself. Tumor growth of rhabdomyosarcoma xenograft in nude mouse model was suppressed by SkQ1. SkQ1 blocked the growth of fibrosarcoma HT1080 and RD tumor cells in culture due to the inactivation of Aurora family kinases (75).

### Mitochondrial Pro-oxidant Strategies

A different anti-tumor redox approach lies in selectively activating mitochondrial oxidative stress in tumor cells by benefitting of their commonly higher mROS levels and abundant antioxidant defense strategy.

#### Mito-Metformin

Drugs for oxidative phosphorylation inhibition like Mito-metformin are able to decrease mitochondrial respiration thanks to its action on the activity of electron transport chain complex I. An interesting proof of action of such molecule has been investigated in pancreatic ductal adenocarcinoma, where Mito-metformin inhibits cancer cell proliferation by enhancing the formation of superoxide and other oxidants, presumably acting on AMPK which, in turn, leads to the ATP depletion and growth arrest. Although it still needs further studies, it is conceivable that Mito-metformin acts via mROS-AMPK axis on AKT/FOXO3/FOXM1 signaling pathway (68). In cancer cells, Mito-metformin reduces hypoxic activation of HIF1 with consequently reduction of cellular proliferation and increase of cell death (76). Tumor cells conveying oncogenic mutations in isocitrate dehydrogenase 1 and 2 (IDH1/2) presented heightened demand on oxidative metabolism and their sensitivity to pharmacological inhibition of OXPHOS is increased (77).

#### MitoVitE and ME-344

MitoVitE (mitotocopherol) consists of TPP adjoined to the a-tocopherol moiety of vitamin E with two carbon chain. MitoVitE debilitates mitochondrial complex I which results in the leakage of electrons and production of mROS and reduces the proliferation of MYC-dependent osteogenic sarcoma cells (78). CSCs are defined by an extremely high plastic metabolism, which in turn allows them to endure and withstand stress circumstances by shifting between glycolysis and OXPHOS (79). Hence, the pharmacological hindrance of oxidative phosphorylation by mitochondrial targeting drugs may block this rescue system of CSCs and would make CSCs more responsive to chemotherapeutics generated to kill the glycolytic cancer cells with high rate of proliferation. Suppression of glycolysis or blocking OXPHOS only has restricted effects on cancer CSCs. The depletion of intracellular ATP (79) occurs only through the combination of the direct targeting of both pathways. A very powerful interdependent result able to counter this resistance was accomplished by an alliance of chemotherapeutics with the inhibitors of mitochondria complex I ME-344 (67), which prevented the change to mitochondrial dependent metabolism. The vitamin E analog, α-tocopheryl succinate mitochondrial-targeted derivative (MitoVES), is a promising mROS targeted anti-cancer drugs with the potential to eliminate CSCs (72).

#### Mito-CP and Mito-CP-AC

Alternative anticancer redox strategies include the use of synthetic mitochondria-targeted inhibitors of complex I, like Mito-CP and Mito-CP-AC to inhibit tumor cell growth and promote cancer cells apoptosis. Mito-CP and Mito-CP analog, Mito-CP-Ac, that incorporate an acetamide group instead of the nitride of Mito-CP, are 10-carbon side chain having a nitride group that can operate stimulation mitochondrial ROS through the inhibition of complex I. The synergism of both Mito-CP and Mito-CP-Ac with 2-deoxy-glucose (2-DG) to deplete intracellular ATP, prevent cell proliferation and cause apoptosis in pancreatic cancer cells. Changing the mitochondrial function and the intracellular citrate levels by mitochondrial antioxidants Mito-CP and Mito-CP-Ac lead to a decline of cell proliferation and the beginning of apoptosis in pancreatic cancer cells (70).

#### Menadione-Ascorbate and β-phenylethylisothiocyanate (PEITC)

Specific molecules that selectively induce mitochondrial oxidative stress are under study. The Menadione-ascorbate combination and PEITC are two promising compounds for this particular purpose. Ascorbate improves menadione redox capacity, advancing to the formation of intracellular mROS (71). Growing the cells in the presence of both ascorbate, menadione and aminotriazole, a catalase inhibitor, causes a great diminishing of cell survival which strengthens the role of H_2_O_2_ as the principal oxidizing agent that kill K562 cells (80, 81). It was demonstrated that in Bcr-Abl expressing hematopoietic cells and in H-RasV12 mutated ovarian epithelial cells these oncogenic transformation lead to mROS generation and contributes to the malignant cells becoming highly sensitive to PEITC, which completely cripples the glutathione antioxidant system and causes severe mROS build-up preferentially in the cancer cells due to their active mROS output (82).

#### Dichloroacetate (DCA)

DCA, a PDK1 inhibitor, is alternative antioxidant that target mitochondria that causes an oxidative-dependent apoptosis in multiple glioblastoma cell lines (73).

### Alternative Mitochondrial ROS Targeted Strategies

#### One Carbon Metabolism Targeting

In order to maintain various antioxidant defense systems, NADPH is fundamental. The production of NADPH in the mitochondria occurs from one carbon metabolism (83), and one carbon metabolism in the mitochondria commences by serine catabolism by serine hydroxymethyltransferase (SHMT2). As indicated in a study, under hypoxia SHMT2 is basic for mitochondrial redox balance in particularly in Myc-transformed cells. This is a characteristic metabolic feature shared by many tumors (33). It has been demonstrated that the reduction of cellular NADPH:NADP^+^ ratio occurs in cells in which SHMT2 knockdown was performed. In these cells, an increase in hypoxia induced cell death is observed. As a result, the tumor growth was reduced by diminishing SHTM2 (33). A strong inhibition of hepatocellular carcinoma xenograft growth using inducible shRNA against SHMT2 has been identified (84). Cancer and normal cells show different expression of methylenetetrahydrofolate dehydrogenase (MTHFD2), a crucial enzyme in the mitochondrial one carbon metabolism (85). The decrease of MTHFD2 leads to an increase of mROS levels sensitizing cancer cells to oxidant-induced cell death, with no effects on normal proliferating cells not expressing the enzyme (85). These are the reasons MTHFD2 may symbolize a likely mitochondrial redox anticancer therapeutic agent. In a murine model of AML, blocking MTHFD2 by silencing with shRNA the animals still succumbed to their disease (85).

#### Mitochondrial Redoxins Targeting

Some studies have found large presence of the antioxidants thioredoxin reductase (TrxR), particularly the mitochondrial thioredoxin (TrxR2), in various types of cancer and this expression has been associated to tumor aggressiveness (86). Similarly, mitochondrial glutaredoxin-2 (Grx2) has been shown to have an anti-apoptotic outcome in cancer cells (87). Additionally, it has been well-established that when glutathione levels decrease in murine breast cancer do not hinder tumor growth but rises Trx activity as a compensatory switch to buffer mROS levels (88). The ability for cancer cells to withstand and adapt to glutathione inhibition by enhancing antioxidant functions of the mitochondria is conveyed in this last data. Targeting TrxR2 had demonstrated to be beneficial in inhibiting multiple myeloma growth by reducing the proteasome function and enhancing cytotoxic oxidative stress (89). Furthermore, Grx2 down regulation may make cells more sensitive to the toxic properties of chemotherapy agents (90).

#### Mitochondrial ROS Generators and PD-1 Immunotherapy

Cancer treatment has been completely influenced by immunotherapy by PD-1 blockades through its long-lasting effect and high efficacy against a wide variety of cancers with limited antagonistic effects (91). Nevertheless, the percentage of patients that still remain insensitive or less responsive to the PD-1 blockade therapy is around 50%. To change and improve upon this lack of response, PD-1 blockade therapy has joined with several other types of cures. Research has shown that mROS generation by molecules that generate mROS (Luperox, chaetocin phytol) or secondarily by uncouplers of mitochondria, carbonylycyanide-p-trifluoromethoxphenylhydrazone (FCCP) and 2, 4-Dinitrophenol (DNP), increases the cancer killing activity of PD-1 blockade by growth of the effector or memory CTLs in draining the lymph node (DLNs) and inside the cancer tissues. It has been shown that tumor reactive cytotoxic T lymphocytes (TR CTLs) treated with PD-L1 may bring triggered mitochondria resulting in higher mROS production. Furthermore, it was revealed that the administration of ROS producers together with uncouplers has strong effects with PD-1 blockade on tumor growth inhibition. The ability of uncouplers to synergize the cytotoxic effects of PD-L1 therapy may be due to their capacity to cause cellular mROS through hypoxia (92).

#### Photo-Thermal and Photo-Dynamic Therapy

Two new strategies in mitochondrial targeting redox cancer therapy are photo-thermal and photo-dynamic therapy. It is possible to bind chemically gold, platinum, carbon, iron, titanium oxide nanoparticles to organic compounds like cyanidines and then connected to TPP, with the goal of selectively store within mitochondria. These nanomaterials and molecules directly or indirectly produce mROS when photo irradiated and this leads to activation of apoptosis which may include mitophagy (93). Some examples used in these therapies are Mito-CCy, mito-CIO, protein-ruthenium hybrids, and FDA approved green indocyanine (94, 95).

## Concluding Remarks

A different method for curing and preventing cancer may lie in mitochondrial redox based treatments, even in combination with chemo and radio therapy. Nonetheless, there are many aspects that influence the effectiveness of the treatment, such as the micro-environment, the stage of the tumor, the antioxidant specific mechanisms of action and the mROS levels within the tumor. In conclusion, mROS and mROS “scavenging” structures which simultaneously target the tumors, may bring about anti-cancer responses thus overcoming malignant transformation and further cancer growth. Reducing the levels of mROS impedes the survival signaling, whereas blocking the cancer's cell antioxidant strategies causes cell death. Therefore, in deciding whether to use a pro-oxidant or anti-oxidant approach for tumor therapy, the definition of a “redox signaling hallmark,” composed of various constraints including the cell redox grade, definition of antioxidants, a signature of cell signaling and transcription factor activation, may be fundamental.

Mitochondrial redox signaling and management are crucial for most hallmarks of cancer, such as initiation and growth, anoikis resistance, stemness achievement, metastasization, and establishment of an immunosuppressive environment. The mROS-dependent effects on several hallmarks of cancer are summarized in the specific boxes.

Cell autonomous include mutations and damage in ETC complexes (Complex I and III) and in mtDNA, leading to a dysfunctional mitochondria highly-producing ROS. Cancer cells harboring mutations in specific TCA cycle enzymes display high levels of mROS. Particularly, FH loss induces fumarate accumulation leading to the succination of GSH and the inactivation of Nrf2- antioxidant response. Nrf2 is a transcription factor involved in the activation of the antioxidant response to combat cytosolic and mitochondrial ROS, upon the release of the repressive molecule Keap1 from Nrf2. In parallel, PGC-1α is a multifunctional transcriptional coactivator controlling mitochondrial dynamics and biogenesis as well as the anti-mROS defense system.

Cell non-autonomous mechanisms involve the tumor cell mitochondrial deregulation/overexploitation induced by stroma. Stromal environment is capable to transfer mtDNA into tumor cells, thus restoring respiration and amplifying mROS-dependent signaling. Also, damage such as radiation promotes mROS-dependent activation of fibroblasts into CAFs, expressing a-SMA. CAFs in turn produce high amount of lactate which—upon imported in cancer cells—fuels TCA cycle (PGC-1α activation is needed for this reprogramming), leading to high levels of signaling-associated mROS, thereby activating Src/Pyk2, Src/PKM2-dependent motility pathways and stabilizing HIF1. Finally, tumor immune environment faces mROS in the events of apoptosis and exhaustion of Treg and effector cells, respectively, thus supporting the tumor immunosuppression. Also, mROS levels are finely modulated for dendritic cells antigen cross-presentation.

## Author Contributions

LI, EG, and MP wrote, elaborated the figure, and critically reviewed the manuscript. PC and MP designed and directed manuscript.

### Conflict of Interest

The authors declare that the research was conducted in the absence of any commercial or financial relationships that could be construed as a potential conflict of interest.
